# Supplementary material for: Estimating risks of importation and local transmission of Zika virus infection
Source: PeerJ. 2016 Apr 5;4:e1904. doi: 10.7717/peerj.1904 (PMC4824915; doi:10.7717/peerj.1904)
Supplement: Supplemental Information 1 [file peerj-04-1904-s001.docx]

Supplementary Materials:

| **Country** | **Reference** |
| --- | --- |
| Uganda | Dick GW, Zika virus. II. Pathogenicity and physical properties. Trans R Soc Trop Med Hyg. 1952:46: 521–534. doi: 10.1016/0035-9203(52)90043-6 |
| Tanzania | Smithburn KC. Neutralizing antibodies against certain recently isolated viruses in the sera of human beings residing in East Africa.J Immunol.1952;69(2):223-34. |
| Indonesia | Olson JG, Ksiazek TG, Suhandiman, Triwibowo. Zika virus, a cause of fever in Central Java, Indonesia. Trans R Soc Trop Med Hyg. 1981;75: 389–393. |
| Malaysia | Marchette NJ, Garcia R, Rudnick A. Isolation of Zika virus from Aedes aegypti mosquitoes in Malaysia. Am J Trop Med Hyg. 1969;18(3):411-5. |
| India | Smithburn KC, Kerr JA, Gatne PB. Neutralizing antibodies against certain viruses in the sera of residents of India. J Immunol. 1954 ;72(4):248-57. |
| Philippines | Hammon WM, Schrack WD Jr, Sather GE. Serological survey for a arthropod-borne virus infections in the Philippines. Am J Trop Med Hyg. 1958;7(3):323-8. |
| Egypt | Smithburn KC, Taylor RM, Rizk F, Kader A. Immunity to certain arthropod-borne viruses among indigenous residents of Egypt. Am J Trop Med Hyg. 1954;3: 9–18. |
| Angola | Kokernot RH, Casaca VM, Weinbren MP, McIntosh BM.Survey for antibodies against arthropod-borne viruses in the sera of indigenous residents of Angola. Trans R Soc Trop Med Hyg. 1965;59(5):563-70. |
| Kenya | Henderson BE, Metselaar D, Cahill K, Timms GL, Tukei PM, Williams MC. Yellow fever immunity surveys in northern Uganda and Kenya and eastern Somalia, 1966-67. Bull World Health Organ.1968;38(2):229-37. |
| Thailand | William L. Pond. Arthropod-borne virus antibodies in sera from residents of South-East Asia. Trans R Soc Trop Med Hyg. 1963; 57(5):364-71 |
| Vietnam | William L. Pond. Arthropod-borne virus antibodies in sera from residents of South-East Asia. Trans R Soc Trop Med Hyg. 1963; 57(5):364-71 |
| Ethiopia | Henderson BE, Tukei PM, Sekyalo E, Mukuye A, Mujomba E. Arbovirus Serological Survey. In: Virus Research Institute Annual Report, East African Printer, Nairobi, Kenya. 1967. pp 29–32. |
| Somalia | Henderson BE, Metselaar D, Cahill K, Timms GL, Tukei PM, Williams MC. Yellow fever immunity surveys in northern Uganda and Kenya and eastern Somalia, 1966-67. Bull World Health Organ.1968;38(2):229-37. |
| Gabon | Saluzzo JF, Ivanoff B, Languillat G, Georges AJ. Serological survey for arbovirus antibodies in the human and simian populations of the South-East of Gabon (author's transl). Bull Soc Pathol Exot Filiales. 1982;75(3):262-6. [Article in French] |
| Nigeria | Moore DL, Causey OR, Carey DE, Reddy S, Cooke AR, Akinkugbe FM, et al. Arthropod-borne viral infections of man in Nigeria, 1964–1970. Ann Trop Med Parasitol 1975;69:49–64. |
| Central African Republic | Faye O, Dupressoir A, Weidmann M, Ndiaye M, Alpha Sall A. One-step RT-PCR for detection of Zika virus. J Clin Virol. 2008;43: 96–101. |
| Senegal | Faye O, Dupressoir A, Weidmann M, Ndiaye M, Alpha Sall A. One-step RT-PCR for detection of Zika virus. J Clin Virol. 2008;43: 96–101. |
| Sierra Leone | Robin Y, Mouchet J. Serological and entomological study on yellow fever in Sierra Leone. Bull Soc Pathol Exot Filiales. 1975;68: 249–258. |
| Pakistan | Darwish MA, Hoogstraal H, Roberts TJ, Ahmed IP, Omar F. A sero-epidemiological survey for certain arboviruses (Togaviridae) in Pakistan. Trans R Soc Trop Med Hyg. 1983;77(4):442-5. |
| Cote d'Ivoire | Faye O, Dupressoir A, Weidmann M, Ndiaye M, Alpha Sall A. One-step RT-PCR for detection of Zika virus. J Clin Virol. 2008;43: 96–101. |
| Burkina Faso | Institut Pasteur de Dakar. WHO collaborating center for reference and research on arboviruses and hemorrhagic fever viruses: Annual report. 1999. Dakar, Senegal. 143 p. |
| Micronesia | Duffy MR, Chen TH, Hancock WT, Powers AM, Kool JL, Lanciotti RS, et al. Zika virus outbreak on Yap Island, Federated States of Micronesia. N Engl J Med. 2009;360(24):2536-43. doi: 10.1056/NEJMoa0805715. |
| United States | Foy BD, Kobylinski KC, Chilson Foy JL, Blitvich BJ, Travassos da Rosa A, Haddow AD, et al. Probable non-vector-borne transmission of Zika virus, Colorado, USA. Emerg Infect Dis. 2011;17(5):880-2. doi: 10.3201/eid1705.101939. |
| Cameroon | Fokam EB, Levai LD, Guzman H, Amelia PA, Titanji VP, Tesh RB, et al. Silent circulation of arboviruses in Cameroon. East Afr Med J. 2010;87(6):262-8. |
| Cambodia | Heang V, Yasuda CY, Sovann L, Haddow AD, Travassos da Rosa AP, Tesh RB, et al. Zika virus infection, Cambodia, 2010. Emerg Infect Dis. 2012;18(2):349-51. doi: 10.3201/eid1802.111224 |
| Australia | Kwong JC, Druce JD, Leder K. Zika virus infection acquired during brief travel to Indonesia. Am J Trop Med Hyg. 2013;89(3):516-7. doi: 10.4269/ajtmh.13-0029. |
| Canada | Fonseca K, Meatherall B, Zarra D, Drebot M, MacDonald J, Pabbaraju K, et al. First case of Zika virus infection in a returning Canadian traveler. Am J Trop Med Hyg. 2014;91(5):1035-8. doi: 10.4269/ajtmh.14-0151. |
| Germany | Tappe D, Rissland J, Gabriel M, Emmerich P, Gunther S, Held G, et al. First case of laboratory-confirmed Zika virus infection imported into Europe, November 2013. Euro Surveill. 2014;19(4). pii: 20685. |
| French Polynesia | Hancock WT, Marfel M, Bel M. Zika virus, French Polynesia, South Pacific, 2013. Emerg Infect Dis. 2014;20(11):1960. doi: 10.3201/eid2011.141380 |
| Japan | Kutsuna S, Kato Y, Takasaki T, Moi M, Kotaki A, Uemura H, et al. Two cases of Zika fever imported from French Polynesia to Japan, December 2013 to January 2014 . Euro Surveill. 2014;19(4). pii: 20683. |
| Noway | Wæhre T, Maagard A, Tappe D, Cadar D, Schmidt-Chanasit J. Zika virus infection after travel to Tahiti, December 2013. Emerg Infect Dis. 2014;20(8):1412-4. doi: 10.3201/eid2008.140302. |
| Italy | Zammarchi L, Stella G, Mantella A, Bartolozzi D, Tappe D, Günther S,et al. Zika virus infections imported to Italy: clinical, immunological and virological findings, and public health implications. J Clin Virol. 2015;63:32-5. doi: 10.1016/j.jcv.2014.12.005. |
| New Caledonia | World Health Organization Western Pacific Region (WPRO). Pacific syndromic surveillance report. Retrieved from: http://www.wpro.who.int/southpacific/en/ (Last accessed: Jan 31, 2016). |
| Cook Islands | World Health Organization Western Pacific Region (WPRO). Pacific syndromic surveillance report. Retrieved from: http://www.wpro.who.int/southpacific/en/ (Last accessed: Jan 31, 2016). |
| Solomon Islands | World Health Organization Western Pacific Region (WPRO). Pacific syndromic surveillance report. Retrieved from: http://www.wpro.who.int/southpacific/en/ (Last accessed: Jan 31, 2016). |
| Zambia | Babaniyi OA, Mwaba P, Songolo P, Mazaba-Liwewe ML, MweeneNdumba I, et al. Seroprevalence of Zika virus infection specific IgG in Western and North-Western Provinces of Zambia. International Journal of Public Health and Epidemiology. 2015;4(1). ISSN: 2326-7291 |
| Belgium | ProMED Mail. http://www.promedmail.org/ (Last accessed on: 31 Jan 2016). |
| Vanuatu | World Health Organization Western Pacific Region (WPRO). Pacific syndromic surveillance report. Retrieved from: http://www.wpro.who.int/southpacific/en/ (Last accessed: Jan 31, 2016). |
| Brazil | Campos GS, Bandeira AC, Sardi SI. Zika Virus Outbreak, Bahia, Brazil. Emerg Infect Dis. 2015;21(10):1885-6. doi: 10.3201/eid2110.150847. |
| Sweden | Anonymous: Swedish tourist got Zika virus on visit to Brazil. Sweden New. Net. 2016  (available from:[http://www.thelocal.se/20160127/swedish-tourist-got-zika-virus-on-visit-to-brazil]) (Last accessed on: 31 Jan 2016). |
| Fiji | World Health Organization Western Pacific Region (WPRO). Pacific syndromic surveillance report. Retrieved from: http://www.wpro.who.int/southpacific/en/ (Last accessed: Jan 31, 2016). |
| Samoa | World Health Organization Western Pacific Region (WPRO). Pacific syndromic surveillance report. Retrieved from: http://www.wpro.who.int/southpacific/en/ (Last accessed: Jan 31, 2016). |
| Colombia | World Health Organization (WHO). Emergencies preparedness, response. Retrieved from:  http://www.who.int/csr/en/ (Last accessed: Jan 31, 2016). |
| Suriname | World Health Organization (WHO). Emergencies preparedness, response. Retrieved from:  http://www.who.int/csr/en/ (Last accessed: Jan 31, 2016). |
| El Salvador | World Health Organization (WHO). Emergencies preparedness, response. Retrieved from:  http://www.who.int/csr/en/ (Last accessed: Jan 31, 2016). |
| Guatemala | World Health Organization (WHO). Emergencies preparedness, response. Retrieved from:  http://www.who.int/csr/en/ (Last accessed: Jan 31, 2016). |
| Mexico | World Health Organization (WHO). Emergencies preparedness, response. Retrieved from:  http://www.who.int/csr/en/ (Last accessed: Jan 31, 2016). |
| Venezuela | World Health Organization (WHO). Emergencies preparedness, response. Retrieved from:  http://www.who.int/csr/en/ (Last accessed: Jan 31, 2016). |
| Netherlands | ProMED Mail. http://www.promedmail.org/ (Last accessed on: 31 Jan 2016). |
| Panama | World Health Organization (WHO). Emergencies preparedness, response. Retrieved from:  http://www.who.int/csr/en/ (Last accessed: Jan 31, 2016). |
| Paraguay | World Health Organization (WHO). Emergencies preparedness, response. Retrieved from:  http://www.who.int/csr/en/ (Last accessed: Jan 31, 2016). |
| Honduras | World Health Organization (WHO). Emergencies preparedness, response. Retrieved from:  http://www.who.int/csr/en/ (Last accessed: Jan 31, 2016). |
| Cape Verde | World Health Organization (WHO). Emergencies preparedness, response. Retrieved from:  http://www.who.int/csr/en/ (Last accessed: Jan 31, 2016). |
| Spain | Anonymous: Spain urges calm amid fears over head-shrinking Zika virus. THE LOCAL. 2016 (available from: [http://www.thelocal.es/20160127/spain-calls-for-calm-amid-head-shrinking-zika-virus-fears]) (Last accessed on: 31 Jan 2016). |
| Puerto Rico | World Health Organization (WHO). Emergencies preparedness, response. Retrieved from:  http://www.who.int/csr/en/ (Last accessed: Jan 31, 2016). |
| Martinique | World Health Organization (WHO). Emergencies preparedness, response. Retrieved from:  http://www.who.int/csr/en/ (Last accessed: Jan 31, 2016). |
| French Guiana | World Health Organization (WHO). Emergencies preparedness, response. Retrieved from:  http://www.who.int/csr/en/ (Last accessed: Jan 31, 2016). |
| United Kingdom | Anonymous: First cases of Zika virus in Europe reported in UK. Daily Sabah Europe. 2016. (available from: [http://www.dailysabah.com/europe/2016/01/24/first-cases-of-zika-virus-in-europe-reported-in-uk]) (Last accessed on: 31 Jan 2016). |
| Taiwan | Centers for Disease Control, R.O.C. (available from: [http://www.cdc.gov.tw/english/info.aspx?treeid=bc2d4e89b154059b&nowtreeid=ee0a2987cfba3222&tid=31FD076D51DBC5BB]) (Last accessed on: 31 Jan 2016). |
| Finland | Korhonen EM, Huhtamo E, Smura T, Kallio-Kokko H, Raassina M, Vapalahti O.　 Zika virus infection in a traveller returning from the Maldives, June 2015.　Euro Surveill. 2016;21(2). doi: 10.2807/1560-7917.ES.2016.21.2.30107. |
| Ecuador | World Health Organization (WHO). Emergencies preparedness, response. Retrieved from:  http://www.who.int/csr/en/ (Last accessed: Jan 31, 2016). |
| Haiti | World Health Organization (WHO). Emergencies preparedness, response. Retrieved from:  http://www.who.int/csr/en/ (Last accessed: Jan 31, 2016). |
| Guadeloupe | World Health Organization (WHO). Emergencies preparedness, response. Retrieved from:  http://www.who.int/csr/en/ (Last accessed: Jan 31, 2016). |
| Barbados | World Health Organization (WHO). Emergencies preparedness, response. Retrieved from:  http://www.who.int/csr/en/ (Last accessed: Jan 31, 2016). |
| Bolivia | World Health Organization (WHO). Emergencies preparedness, response. Retrieved from:  http://www.who.int/csr/en/ (Last accessed: Jan 31, 2016). |
| Guyana | World Health Organization (WHO). Emergencies preparedness, response. Retrieved from:  http://www.who.int/csr/en/ (Last accessed: Jan 31, 2016). |
| Argentina | Rizzi M, Lough R, Dunham W: Argentina confirms first Zika virus case - health ministry sources. The Star Online. (available from: [http://www.thestar.com.my/news/world/2016/01/29/argentina-confirms-first-zika-virus-case--health-ministry-sources/]) (Last accessed on: 31 Jan 2016). |
| Peru | Taj M, Baum B: Peru reports first patient carrying the Zika virus. REUTERS. (available from: [http://www.reuters.com/article/us-health-zika-peru-idUSKCN0V71XA]) (Last accessed on: 31 Jan 2016). |
| Portugal | Anonymous: EU on high alert in response to first cases of Zika virus. Finland Times.  [http://www.reuters.com/article/us-health-zika-peru-idUSKCN0V71XA]) (Last accessed on: 31 Jan 2016). |
| Austria | Anonymous: First confirmed case of Zika virus in Austria. THE LOCAL  [http://www.thelocal.at/20160128/first-confirmed-case-of-zika-virus-in-austria]) (Last accessed on: 31 Jan 2016). |
| Costa Rica | Dyer Z: First Zika virus case reported in Costa Rica. The Tico Times News.  [http://www.ticotimes.net/2016/01/26/first-zika-virus-case-reported-costa-rica]) (Last accessed on: 31 Jan 2016). |
| Switzerland | Anonymous: More Zika cases found in European tourists returning from South America. Jamaica Observer. 2016. (available from: [http://www.jamaicaobserver.com/latestnews/More-Zika-cases-found-in-European-tourists-returning-from-South-America]) (Last accessed on: 31 Jan 2016). |
| Dominican Republic | World Health Organization (WHO). Emergencies preparedness, response. Retrieved from:  http://www.who.int/csr/en/ (Last accessed: Jan 31, 2016). |
| Jamaica | Anonymous: Jamaica confirms first case of Zika virus. CTV News. 2016  (available from: [http://www.ctvnews.ca/health/jamaica-confirms-first-case-of-zika-virus-1.2758579]) (Last accessed on: 31 Jan 2016). |
| Denmark | Anonymous: Zika virus spreading in Europe as 1st person tests positive in Denmark. 2016. (available from: [https://www.rt.com/news/330262-zika-virus-strikes-europe/]) (Last accessed on: 31 Jan 2016). |
| Virgin Islands | World Health Organization (WHO). Emergencies preparedness, response. Retrieved from:  http://www.who.int/csr/en/ (Last accessed: Jan 31, 2016). |
| Nicaragua | Herriman R. : Nicaragua and Curacao confirm local transmission of Zika (available from: [http://outbreaknewstoday.com/nicaragua-and-curacao-confirm-local-transmission-of-zika-12207/]) (Last accessed on: 31 Jan 2016). |
| Tonga | World Health Organization (WHO). Emergencies preparedness, response. Retrieved from:  http://www.who.int/csr/en/ (Last accessed: Jan 31, 2016). |
